# Spatial distribution-based progression of spinal cord injury pathology: a key role for neuroimmune cells

**DOI:** 10.3389/fimmu.2024.1505755

**Published:** 2025-01-09

**Authors:** Jian Li, Xiaolei Zhai, Chaochun Yu

**Affiliations:** ^1^ Shanghai TCM-Integrated Hospital, Shanghai University of TCM, Department of Neurosurgery, Shanghai, China; ^2^ Department of Neurosurgery, Shuyang Hospital of Traditional Chinese Medicine, Affiliated Shuyang Hospital of Nanjing University of Chinese Medicine, Shuyang, China

**Keywords:** spinal cord injury, pathology, neuroimmune cells, progression, spatial distribution, immune cells, inflammatory response, immunomodulation

## Abstract

An external trauma, illness, or other pathological cause can harm the structure and function of the spinal cord, resulting in a significant neurological disorder known as spinal cord injury (SCI). In addition to impairing movement and sensory functions, spinal cord injury (SCI) triggers complex pathophysiological responses, with the spatial dynamics of immune cells playing a key role. The inflammatory response and subsequent healing processes following SCI are profoundly influenced by the spatial distribution and movement of immune cells. Despite significant advances in both scientific and clinical research, SCI therapy still faces several challenges. These challenges primarily stem from our limited understanding of the spatial dynamics of immune cell distribution and the processes that regulate their interactions within the microenvironment following injury. Therefore, a comprehensive investigation into the spatial dynamics of immune cells following SCI is essential to uncover their mechanisms in neuroinflammation and repair, and to develop novel therapeutic strategies.

## Introduction

Spinal Cord Injury (SCI) is a severe neurological condition that causes both structural and functional damage to the spinal cord. It is most commonly induced by trauma or other pathological factors (1–5). The likelihood of long-term functional recovery is primarily influenced by secondary pathological processes that occur subsequent to the initial physical injury, thereby contributing to the complexity of SCI (6–9). In these secondary processes, the immune system—specifically neuroimmune cells—plays a critical role. Through both inflammatory and reparative mechanisms, these cells significantly influence the overall progression of SCI. Consequently, a comprehensive understanding of the spatial distribution and dynamic behavior of immune cells following SCI is crucial for the creation of efficacious treatment approaches (10–12).

## Immune cells and their early role in spinal cord injury

In the early stages of Spinal Cord Injury (SCI), both systemic and local immune responses are rapidly activated, initiating a complex cascade of pro- and anti-inflammatory activities (7, 13–15). Neutrophils are among the first immune cells to reach the site of injury during these events, with chemokines such as CXCL1 and CXCL2 playing a key role in regulating their migration. Through interaction with the CXCR2 receptor, these chemokines drive the chemotactic movement of neutrophils toward the damaged tissue (16–20). Neutrophils play a crucial role in the early clearance of necrotic tissue and the initiation of the healing process. However, when neutrophils become hyperactivated, they release pro-inflammatory cytokines that exacerbate the inflammatory response, leading to further tissue damage and secondary injury (21–23).

## Regulation of immune response in SCI recovery

As the inflammatory response intensifies, other immune cells, such as T lymphocytes and macrophages, become involved in regulating the surrounding environment (24–27).Macrophages are activated by interferon-γ (IFN-γ) produced by Th1 cells and primarily drive the pro-inflammatory response through the secretion of tumor necrosis factor-α (TNF-α), a potent mediator of inflammation. If not properly regulated, TNF-α can exacerbate tissue damage (28–31). On the other hand, Th2 cells help mitigate excessive immune activity and promote tissue regeneration by secreting anti-inflammatory cytokines, such as IL-4 and IL-10. Therefore, the regulation of both tissue damage and healing processes relies on the balance between Th1 and Th2 cells.

In addition to Th1 and Th2 cells, Th17 cells and regulatory T cells (Tregs) play critical roles in immunological control following SCI (32–34). By secreting IL-17, Th17 cells promote the recruitment of neutrophils during the chronic inflammatory phase, which can lead to prolonged inflammation and exacerbate long-term spinal cord injury. In contrast, Tregs secrete anti-inflammatory molecules, such as IL-10, to suppress the activity of Th1 and Th17 cells, thereby promoting tissue repair and reducing inflammation. The anti-inflammatory properties of Tregs are crucial for controlling hyperactive immune responses and facilitating the repair of damaged tissue.

Furthermore, B cells and natural killer (NK) cells are integral to the complex immune response following spinal cord injury. B cells can both promote and impede tissue healing by secreting cytokines such as IL-1β and IL-6, particularly during the early phases of the repair process. NK cells, through their cytotoxic activity, primarily eliminate damaged cells. While moderate NK cell activity contributes to cell clearance, excessive activation can cause collateral damage to healthy cells, exacerbating injury (35, 36).

The pathological progression of SCI is determined by the dynamic balance between pro-inflammatory and anti-inflammatory responses. Pro-inflammatory signals, including Th1, Th17, and neutrophils, play a critical role in the acute phase by initiating the immune response and facilitating the clearance of damaged tissue (37, 38). On the other hand, prolonged pro-inflammatory responses can lead to chronic inflammation and tissue degradation. In contrast, anti-inflammatory responses, such as those mediated by Th2 cells and Tregs, are essential for controlling inflammation and promoting tissue repair. However, an overly aggressive or premature anti-inflammatory response may hinder the removal of damaged cells, thereby compromising the healing process.

Therefore, to minimize secondary damage and enhance neurological recovery, precise regulation of the immune response’s timing and spatial distribution is crucial in post-SCI therapy (5, 7, 10, 39).

## Immune cell regulatory networks in inflammatory response and repair after spinal cord injury

Spinal cord injury (SCI) rapidly triggers both local and systemic immune responses involving various immune cells and the cytokines they release. These responses coordinate to control inflammation and tissue healing at the injury site.

**Figure 1:**
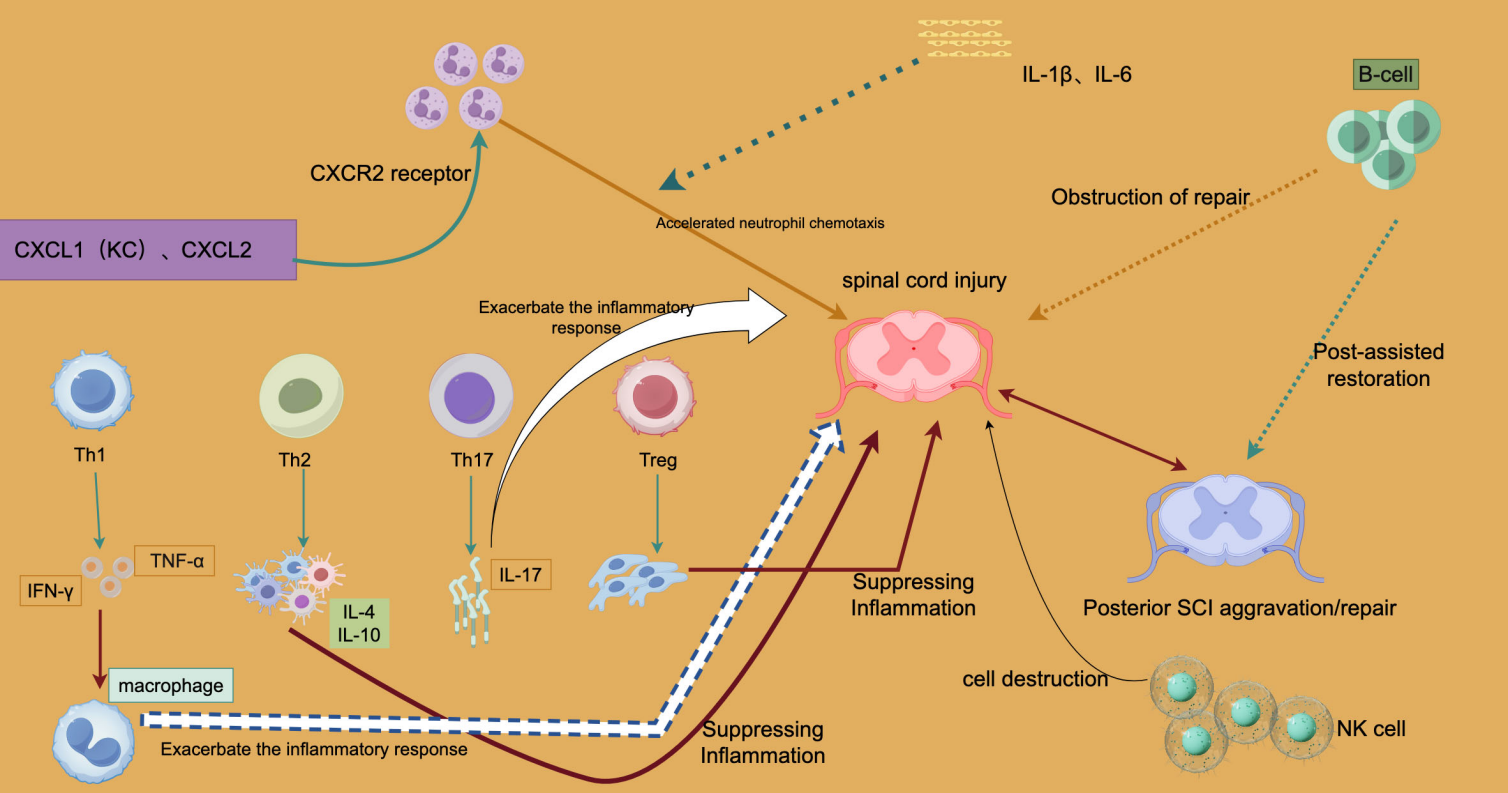
Immune-Regulatory Processes in Spinal Cord Injury Inflammation and Repair.

**Figure 1** illustrates the complex interactions of immune cells following SCI, focusing on how these cells influence injury progression and repair via pro- or anti-inflammatory pathways.

In the early stages of SCI, chemotactic proteins such as CXCL1 (KC) and CXCL2 are upregulated, driving neutrophil chemotaxis through the CXCR2 receptor. This rapid neutrophil influx helps remove necrotic cells and damaged tissue but can also lead to an excessive production of pro-inflammatory cytokines, worsening tissue damage and the local inflammatory response. This process is depicted in **Figure 1** as “Accelerated neutrophil chemotaxis.”

T cells play a critical role in the immune response following SCI, with subsets including Th1, Th2, Th17, and Treg cells. Each subset contributes to inflammation or repair through the secretion of specific cytokines. For example, Th1 cells release interferon-γ (IFN-γ), triggering macrophages to produce tumor necrosis factor-α (TNF-α), a strong pro-inflammatory cytokine that exacerbates inflammation and tissue damage in the early stages. In contrast, Th2 cells secrete anti-inflammatory cytokines like IL-10 and IL-4, which suppress pro-inflammatory macrophages and mitigate inflammation, promoting tissue healing in the later phases of SCI. This anti-inflammatory effect is illustrated by the “Suppressing Inflammation” arrow in **Figure 1**.

Th17 cells, through the secretion of IL-17, activate neutrophils and further intensify the inflammatory response, contributing to chronic inflammation and potentially worsening tissue damage in the later stages of SCI. **Figure 1** highlights this by showing how Th17 cells “Exacerbate the inflammatory response.”

Treg cells, key regulators of the immune response, prevent excessive inflammation by suppressing the activation of Th1 and Th17 cells. Their anti-inflammatory action, as shown in **Figure 1**, facilitates tissue repair by maintaining a balance between pro- and anti-inflammatory signals.

NK and B cells also have dual roles in SCI. B cells can impede tissue repair by secreting pro-inflammatory cytokines like IL-1β and IL-6, as indicated by the “Obstruction of repair” caption in **Figure 1**. Although their exact role remains unclear, B cells may contribute to the healing process in later stages of SCI. NK cells, while playing a protective role in clearing damaged cells, can exacerbate injury by causing excessive destruction of both damaged and healthy cells if overactive.

The balance between pro-inflammatory signals (mediated by Th1, Th17, and neutrophils) and anti-inflammatory signals (mediated by Th2, Treg cells, and other factors) determines the immune response outcome following SCI. Early pro-inflammatory responses are crucial for immunological clearance, but their persistence can lead to chronic inflammation and tissue damage. Conversely, premature or excessive anti-inflammatory responses may hinder early tissue repair and prevent complete recovery.

**Figure 1** also illustrates two possible outcomes in the later stages of SCI: exacerbation or repair (Posterior SCI Aggravation/Repair). This distinction highlights how the inflammatory response following SCI directly impacts long-term recovery. Persistent pro-inflammatory signals can lead to chronic inflammation and further tissue damage, while dominance of anti-inflammatory responses promotes tissue repair, though this process may still be hindered by NK and B cells.

Overall, **Figure 1** reveals the complex regulatory mechanisms of the immune response after SCI. Neutrophil chemotaxis driven by CXCL1 and CXCL2 exacerbates inflammation; Th1 and Th17 cells worsen both the acute and chronic phases of injury by secreting pro-inflammatory cytokines like IFN-γ, TNF-α, and IL-17. In contrast, Th2 and Treg cells mitigate inflammation and support repair by secreting anti-inflammatory cytokines. Additionally, B cells and NK cells play dual roles, either worsening inflammation or contributing to late-stage repair.

## The NLRP3 inflammatory vesicle activation and microglia reactivation pathway are mediated by DAMPs

Focusing on danger-associated molecular patterns (DAMPs), TLR4 receptors, P2X7 receptors, NLRP3 inflammasomes, and pro-inflammatory mediators, this mechanistic model highlights key signaling pathways involved in the immune response. These mechanisms play a crucial role in the regulation of inflammation and microglial activation.

DAMPs, such as ATP and HMGB1, are released from injured or stressed cells and bind to the pattern-recognition receptor TLR4. This binding activates a MyD88-dependent signaling cascade, which promotes the production of downstream inflammatory mediators by phosphorylating IκB and activating nuclear factor κB (NF-κB). Additionally, ATP interacts with the P2X7 receptor, a ligand-gated ion channel essential for the formation and initiation of NLRP3 inflammasomes. NLRP3, activated by alterations in the intracellular environment, facilitates the cleavage of pro-inflammatory precursors into their active forms, such as IL-1β and IL-18. These cytokines effectively activate peripheral immune cells, amplifying the inflammatory response. Through the MAPK pathway, TLR4 signaling further enhances the synthesis of inflammatory factors, intensifying local inflammation and recruiting additional immune cells.

**Figure 2:**
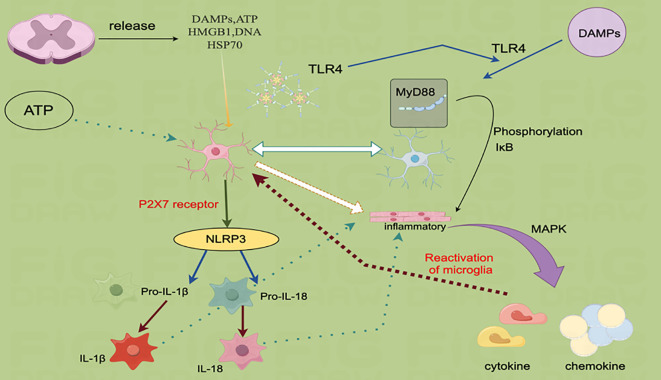
The NLRP3 inflammatory vesicle activation and microglia reactivation pathway are mediated by DAMPs.

**Figure 2** illustrates the process of microglial reactivation, where ATP and DAMPs stimulate microglia to produce inflammatory mediators, contributing to chronic neuroinflammation seen in conditions like Parkinson’s and Alzheimer’s disease. This diagram summarizes how DAMPs reactivate microglia and amplify the inflammatory response by activating NLRP3 through TLR4 and P2X7 receptors. By emphasizing the significance of these pathways in inflammation and neurological disorders, understanding them may pave the way for the identification of novel therapeutic targets for inflammation-related diseases.

## Keyword relationships among published publications in this field of study

**Figure 3:**
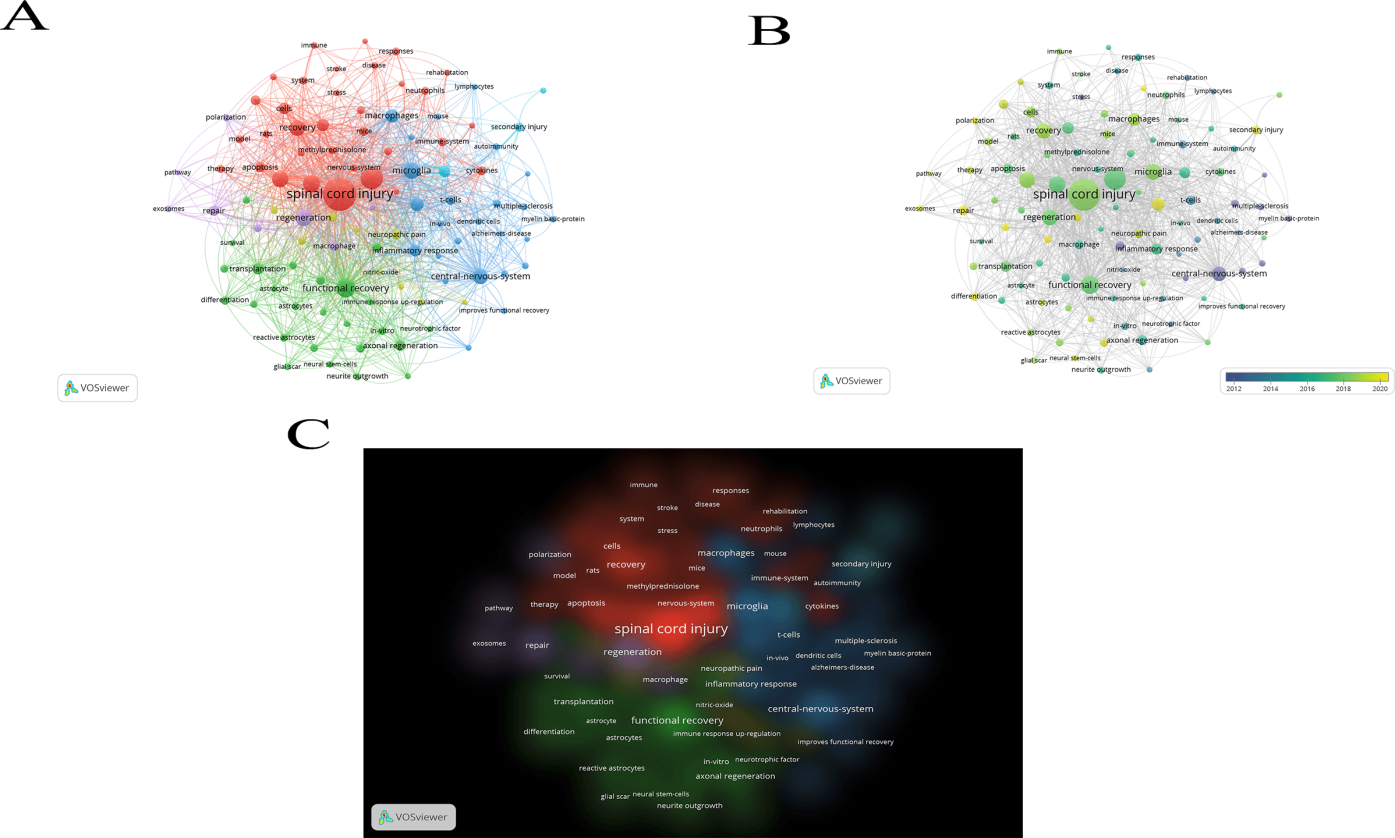
Relationship between keywords of published articles in this field of research.

The field of spinal cord injury (SCI) and immune system interactions can be comprehensively understood by analyzing the current research landscape and future trends through a bibliometric approach using the keywords “spinal cord injury” and “immune.” By applying VOSviewer for keyword analysis, we identified emerging research patterns, key areas for future exploration, and potential gaps in the literature. **Figures 3A–C** depicts the connection network graph between keywords, the keyword heat over time graph, and the keyword density graph, respectively, which illustrates the link between keywords of published papers in this field of study. Of these, **Table 1** shows the 10 most common keywords used in research in this area.

**Table 1:** Top 10 most frequent keywords for research in this field.

ID	Keyword	Occurrences	Total link strength
1	spinal cord injury	481	751
2	inflammation	215	443
3	functional recovery	156	316
4	expression	125	277
5	microglia	121	309
6	activation	114	289
7	regeneration	114	264
8	recovery	107	211
9	central-nervous-system	104	184
10	macrophages	81	222

Over the past few decades, significant progress has been made in SCI research. According to bibliometric data, the relationship between immune responses and spinal cord injury has garnered increasing attention, indicating growing interest in this area of study. Research has shown that, following injury, alterations in both local and systemic immune responses play a crucial role in recovery and regeneration. The immune system is a pivotal factor in the pathophysiological response to SCI, and scientists are now exploring ways to modulate immune responses to improve SCI prognosis and outcomes.

Keywords such as “inflammation,” “regeneration,” and “neuroprotection” frequently appear in the study of keyword co-occurrence, indicating that current research is primarily focused on regulating inflammation and promoting neuroregeneration. This suggests that understanding nerve regeneration processes and controlling inflammation are central themes of contemporary research. These studies provide a critical theoretical foundation for the development of innovative therapeutic strategies, such as cell-based therapies to restore nerve function and anti-inflammatory treatments.

Despite the valuable insights gained from previous research, several questions remain unresolved. The immune response following spinal cord injury is a complex process involving the interplay of various immune cells and cytokines. Future research should place greater emphasis on the roles of specific immune cells at different stages post-injury, particularly the overlapping functions of monocytes, macrophages, and lymphocytes during tissue regeneration. Furthermore, with the advancement of precision medicine, high-throughput technologies such as proteomics and genomics could enable the development of personalized immune-modulating therapies by uncovering individual variations in spinal cord injury patients.

The effectiveness of current therapeutic interventions in restoring function after spinal cord injury remains limited. Future efforts should focus on translating basic research findings into practical applications, particularly within the framework of clinical trials for immunomodulators and cell therapies. Achieving this will require multidisciplinary collaboration and continued support for fundamental research to strengthen the connections between neurology, immunology, and regenerative medicine.

Future research on the link between spinal cord injury and immunity, aided by emerging technologies such as stem cell therapy and single-cell sequencing, is expected to yield groundbreaking discoveries. These advances will deepen our understanding of SCI mechanisms and lead to more effective therapies.

Despite considerable progress, many areas of SCI and immune response remain unexplored. Future studies should focus on unraveling these mechanisms and translating this knowledge into clinical treatments for better patient outcomes.

The high frequency of key terms such as “spinal cord injury,” “inflammation,” “immune response,” “repair,” and “glial scarring” highlights the primary focus of current research. “Cytokines” are a major area of study due to their role in the inflammatory response, while “neuroprotective” and “immunomodulatory” strategies have become key research themes. Stem cell therapy has gained attention as a promising regenerative approach, and the development of “biomarkers” is improving injury assessment and treatment efficacy, facilitating personalized care.

From a bibliometric perspective, these trends suggest that the future of SCI research will focus on integrated treatment strategies that combine immunomodulation and regenerative medicine to enhance patient recovery and quality of life.

## Discussion

The immune response to spinal cord injury (SCI) is complex, with tissue recovery heavily influenced by the dynamic distribution and function of immune cells following injury (5, 40–42). Previous research has shown that the aggregation and migration of immune cells, such as neutrophils, T cells, and macrophages, play a crucial role in neuronal repair and regeneration following spinal cord injury (SCI), affecting both the acute and chronic stages.

This study further elucidates the dynamic behavior of various immune cell types in the recovery process by examining their spatial distribution. It reveals that immune cells not only accumulate at the injury site but also spread across the injured spinal cord. This finding raises important questions regarding the mechanisms of secondary damage and the regeneration of distant neurons, suggesting that immune cell activity may extend beyond the immediate injury site, potentially influencing a broader neural network.

Immune cells play dual roles in spinal cord injury (SCI). On the one hand, during the acute phase, various cells, including neutrophils and macrophages, rapidly proliferate to facilitate the removal of pathogens and necrotic tissue, while also helping to prevent infection (15, 43). However, on the other hand, an excessive inflammatory response and prolonged activation of immune cells can exacerbate tissue damage and neuronal death, hindering the healing and regeneration of nerve tissue (44–46).

Studies have shown that there are significant variations in the number and distribution of immune cells at different stages following spinal cord injury (47–49). For example, during the acute phase, macrophages proliferate significantly, but their numbers decrease during the chronic phase. This aligns with recent studies indicating that the intensity of the immune response diminishes over time. This dynamic shift suggests that strategically timing the activation of immune cells could be a potential approach to enhance spinal cord regeneration and functional recovery.

It is important to note that the activation state of immune cells influences their function, in addition to their quantity. For instance, M2-type macrophages promote tissue healing, while M1-type macrophages are primarily involved in mediating inflammatory responses (50, 51). Therefore, modulating the polarization of macrophages could potentially stimulate neuronal regeneration while reducing inflammatory damage. Numerous recent studies support this concept, suggesting it could become a new therapeutic focus for spinal cord injury treatment.

Spatial dynamics analysis of immune cell movement pathways following spinal cord injury has revealed that different immune cell types migrate along distinct routes and at varying speeds (17, 52–55). T cells, for instance, travel more slowly, but they may have a longer-lasting effect on tissue healing (56, 57). Through more detailed spatial distribution analysis, the mechanisms by which these cells interact with neurons, glial cells, etc. can be further revealed.

The spatial dynamics analysis in this study makes a significant contribution by shedding light on the interactions between immune cells in the injured area and the surrounding tissues. This finding underscores the dual regulatory role of immune cells in both spatial and temporal control during spinal cord injury recovery, aligning with the established cell migration hypothesis. It not only deepens our understanding of the functional division of labor among immune cells but also offers new insights for targeted therapeutic strategies. For instance, future research will likely focus on how to precisely modulate the immune response at specific sites through pharmacological or gene therapy.

This research opens up novel therapeutic avenues for spinal cord injury treatment by potentially stimulating neuronal regeneration and reducing secondary damage through precise regulation of immune cell location and activity. Immunomodulatory therapies are currently showing great promise in treating various conditions, such as multiple sclerosis and other neurodegenerative diseases, by modulating T cell and macrophage activity. Consequently, a key area for future research is adapting these approaches for spinal cord injury management.

However, it is important to recognize that each patient’s immune response to spinal cord injury is unique, varying according to the type and severity of the lesion. Therefore, developing personalized treatment plans will be a critical challenge moving forward. Additionally, a crucial focus for future studies will be how to specifically modulate the immune response at the injury site without compromising the overall function of the immune system.

## Conclusion

This study explores the spatial dynamics of immune cells in spinal cord injury, highlighting their complex role in the recovery process. Immune cells not only contribute to healing and protecting the injured site but may also influence a broader area of the spinal cord through their migration. Precise modulation of the spatial distribution and functional state of immune cells, based on deeper mechanistic insights and clinical trials, is expected to lead to more effective treatments for individuals with spinal cord injuries in the future.

Although this article provides a more in-depth analysis of the spatial dynamics of immune cells and their role in the repair process after spinal cord injury, there are still some shortcomings. First, although the article describes the functions and interactions of different immune cells after spinal cord injury, the mechanisms and spatial distribution of immune cell regulation, especially in the chronic phase of the immune response and its effects on nerve repair, are still not fully explored. In addition, the causal relationship between immune cell migration and tissue repair has not been fully understood, and future studies should focus on the mechanisms of time-window regulation of immune cells in different stages of spinal cord injury, especially the role in the transition of immune response between the acute and chronic stages.

Second, although the article mentions the spatial distribution and dynamic behavior of immune cells, it does not delve into the effects of the interaction of these cells in local and distal regions on the recovery of neurological function. The migratory pathways of immune cells and their roles in different regions of spinal cord injury still need to be further clarified, and the mechanisms of interaction between immune cells and neurons, glial cells and other cells in the injury microenvironment still need to be investigated in greater depth. These interaction processes can be further revealed in the future with the help of single-cell RNA sequencing and immunohistochemical staining.
